# Transgender persons’ view on previous fertility decision-making and current infertility: a qualitative study

**DOI:** 10.1093/humrep/deae155

**Published:** 2024-07-15

**Authors:** J D Asseler, I de Nie, F B van Rooij, T D Steensma, D Mosterd, M O Verhoeven, M Goddijn, J A F Huirne, N M van Mello

**Affiliations:** Department of Obstetrics and Gynaecology, Amsterdam UMC, Location Vrije Universiteit Amsterdam, Amsterdam, The Netherlands; Centre of Expertise on Gender Dysphoria, Amsterdam UMC, Amsterdam, The Netherlands; Amsterdam Reproduction and Development Research Institute, Amsterdam, The Netherlands; Department of Obstetrics and Gynaecology, Amsterdam UMC, Location Vrije Universiteit Amsterdam, Amsterdam, The Netherlands; Centre of Expertise on Gender Dysphoria, Amsterdam UMC, Amsterdam, The Netherlands; Amsterdam Reproduction and Development Research Institute, Amsterdam, The Netherlands; Department of Endocrinology, Amsterdam UMC, Amsterdam, The Netherlands; Research Institute Child Development and Education, Faculty of Social and Behavioural Sciences, University of Amsterdam, Amsterdam, The Netherlands; Centre of Expertise on Gender Dysphoria, Amsterdam UMC, Amsterdam, The Netherlands; Department of Medical Psychology, Amsterdam UMC, Amsterdam, The Netherlands; Department of Obstetrics and Gynaecology, Amsterdam UMC, Location Vrije Universiteit Amsterdam, Amsterdam, The Netherlands; Department of Obstetrics and Gynaecology, Amsterdam UMC, Location Vrije Universiteit Amsterdam, Amsterdam, The Netherlands; Amsterdam Reproduction and Development Research Institute, Amsterdam, The Netherlands; Amsterdam Reproduction and Development Research Institute, Amsterdam, The Netherlands; Centre for Reproductive Medicine, Amsterdam UMC, Location University of Amsterdam, Amsterdam, The Netherlands; Department of Obstetrics and Gynaecology, Amsterdam UMC, Location Vrije Universiteit Amsterdam, Amsterdam, The Netherlands; Amsterdam Reproduction and Development Research Institute, Amsterdam, The Netherlands; Department of Obstetrics and Gynaecology, Amsterdam UMC, Location Vrije Universiteit Amsterdam, Amsterdam, The Netherlands; Centre of Expertise on Gender Dysphoria, Amsterdam UMC, Amsterdam, The Netherlands; Amsterdam Reproduction and Development Research Institute, Amsterdam, The Netherlands

**Keywords:** gender-affirming treatment, transgender, infertility, qualitative research, cryopreservation, gender minorities and parenting

## Abstract

**STUDY QUESTION:**

How do adult transgender and gender diverse (TGD) people, who are infertile due to prior gender-affirming treatment, view their current infertility and their reproductive decisions made in the past?

**SUMMARY ANSWER:**

In a time where sterilization was mandatory, transgender adolescents prioritized gender-affirming treatment over their future fertility and would make the same choice today despite emotional challenges related to infertility experienced by some.

**WHAT IS KNOWN ALREADY:**

Under transgender law in the Netherlands, sterilization was required for legal gender recognition until 2014, resulting in permanent infertility. The long-term consequences of this iatrogenic infertility in transgender adolescents who have now reached adulthood remain underexplored.

**STUDY DESIGN, SIZE, DURATION:**

Qualitative study design based on 21 in-depth one-on-one semi-structured interviews.

**PARTICIPANTS/MATERIALS, SETTING, METHODS:**

TGD people in a stage of life where family planning may be a current topic were eligible for participation. They all received gender-affirming treatment in adolescence prior to the legislation change in 2014. A purposeful sampling technique was used from participants of another ongoing study. Eleven people assigned female at birth and ten people assigned male at birth were included. Interview transcripts were thematically analysed using a modified version of Braun and Clarke’s six steps theory.

**MAIN RESULTS AND THE ROLE OF CHANCE:**

Six main themes were generated: (i) personal considerations regarding fertility and fertility preservation in the past; (ii) external considerations regarding fertility and fertility preservation in the past; (iii) current vision on past considerations and decisions; (iv) Current experiences and coping with infertility; (v) future family building; (vi) advice regarding fertility and fertility preservation decision-making.

**LIMITATIONS, REASONS FOR CAUTION:**

Selection, recall, and choice supportive bias may play a role in interpreting our results.

**WIDER IMPLICATIONS OF THE FINDINGS:**

This study highlights the importance of tailored counselling and comprehensive information on fertility preservation for transgender individuals, especially adolescents, undergoing gender-affirming treatment.

**STUDY FUNDING/COMPETING INTEREST(S):**

N/A.

**TRIAL REGISTRATION NUMBER:**

N/A.

## Introduction

People experiencing gender incongruence may seek medical treatment to aid in their gender-affirming transition. This treatment may consist of gender-affirming hormone therapy (GAHT) and/or gender-affirming surgeries (GAS) (Coleman *et al.*, 2022). People assigned male at birth, but do not identify as such, will be referred to as trans feminine people, which includes non-binary people assigned male at birth. People assigned female at birth, but do not identify as such, will be referred to as trans masculine people, which includes non-binary people assigned female at birth. For trans feminine people, GAHT may consist of testosterone blockers and oestrogens. Although GAHT has been shown to improve a person’s quality of life (Nobili *et al.*, 2018; Coleman *et al.*, 2022), this treatment inhibits gamete maturation in trans feminine people, causing a severe to complete reduction of spermatogenesis(Stolk *et al.*, 2023). This negative effect may be reversible but data on the quality of gametes after prolonged GAHT and outcomes of children born from these gametes are sparse (de Nie *et al.*, 2023b). For trans masculine people, GAHT consists of testosterone. Even though testosterone causes amenorrhea in most people, ovulation may still occur and chances of getting pregnant remain (Asseler *et al.*, 2024). Following a cessation period of testosterone, successful oocyte vitrification and healthy pregnancies have been reported (Light *et al.*, 2014; Asseler *et al.*, 2023). GAS may include gonadectomy, causing permanent infertility in all people (Stolk *et al.*, 2023).

From 2000 onward, a new treatment protocol was established in the Netherlands consisting of gonadotropin-releasing hormone agonist (GnRHa) when entering early puberty (≥Tanner Stage 2) which reversibly inhibits the production of sex hormones and prevents further development of secondary sex characteristics. Hereby, adolescents have more time to explore their gender identity and gender-affirming treatment options (Cohen-Kettenis *et al.*, 2008).

Under Dutch transgender law, sterilization was required until 2014 for legal gender recognition. Many transgender and gender diverse (TGD) people therefore underwent treatments resulting in permanent infertility. This reproductive loss was considered an unavoidable consequence of gender-affirming treatment and options for fertility preservation were hardly discussed or offered (de Nie *et al.*, 2023a). Since this law was abolished, a change in protocol has been made and TGD people are counselled regarding the options for fertility preservation before they initiate gender-affirming treatment in line with international guidelines (Coleman *et al.*, 2022).

Data on changing fertility desires and -preservation are scarce, especially when the decision-making took place in adolescence at a time when legislation determined options for fertility preservation. A systematic review by Stolk *et al.* highlights the knowledge gap in fertility research for TGD people. They included many studies on desire for children in TGD people. However, most were cross-sectional questionnaires, case series, or small sample-size cohort studies with limited follow-up time and lack of control groups. Furthermore, the quality assessment of the included studies was low to moderate (Stolk *et al.*, 2023). One quantitative survey study by de Nie *et al.* (2023a), reported on the changing reproductive desires in adult TGD people whom started medical affirmation in adolescence prior to 2014 and are currently infertile. Here, 56% of participants reported to have children, have a current or future desire for children. 31% of study participants indicated to struggle with their iatrogenic infertility in varying degrees (de Nie *et al.*, 2023a).

This current study aims to qualitatively explore the view of adult TGD people, who are infertile due to prior gender-affirming treatment, on their infertility and reproductive decisions in the past and how this influenced their choices regarding family building currently. Overseeing desires for potential family building can be difficult at a young age. Furthermore, people’s desire for parenthood may change in adulthood compared to adolescence, especially when choices in adolescence are influenced by legislation (prior to 2014) and access to desired GAHT.

## Materials and methods

### Study design

This qualitative study is based on in-depth one-on-one semi-structured interviews. Inclusion criteria were people who (I) received gender-affirming treatment in adolescence prior to the legislation change in 2014, (II) are infertile due to set treatment, and (III) are currently in a phase of life where family planning may be a present topic.

Participants were recruited via another ongoing unrelated study on long-term follow-up of GnRHa and GAHT use in adolescence at the Centre of Expertise on Gender Dysphoria in Amsterdam (van der Loos *et al.*, 2023). While scheduling their physical check-up for that study, suitable candidates were asked by recruiters if they were willing to participate in an interview study immediately following their physical, at the same location. A purposeful sampling technique based on assigned sex at birth was used by recruiters to ensure equal distribution of trans masculine and feminine people. Twenty-two people were asked to participate, of which one person declined participation due to the topic of fertility being too painful. Participants received a lunch voucher as compensation for participation. This study was approved by the local Ethical Committee. Written informed consent was obtained from all participants.

### Data generation

The semi-structured interview script covered the following phases: (i) prior/during medical affirmation, (ii) following medical affirmation, and (iii) currently. Core questions were devised by clinical experiences of researchers in different fields of transgender care (endocrine, gynaecology, and psychology). Core questions were explored with each participant and if clarification was needed, supplementary questions were asked. Additional topics were explored based upon the direction of the individual interview. For an overview of the core questions, see Supplementary Table S1.

One-on-one semi-structured interviews were conducted by three trained authors. In total, 21 interviews were conducted, of which 19 were in person and two by telephone during a COVID-19 lockdown. The date and time were decided by mutual agreement and were scheduled on a day the participant had their (bi-)annual hormone check-up. The interviews were conducted in Dutch, between March 2020 and April 2021. The interviews ranged from 13 to 46 min with an average length of 23 min. With oral consent of the participants, the interviews were recorded on an assigned digital voice recorder. After transcribing verbatim, the de-identified recordings and the transcriptions were stored on a secured hard drive at the Amsterdam UMC in accordance with their regulations.

### Data analysis

Due to lack of prior qualitative research in this specific group, no codes or themes were generated prior to data coding and analysis. Braun and Clarke’s six phases of reflexive thematic analyses (TA) was the theoretical framework chosen for our data coding and analysis since it was most applicable to our research question and study methodology with its inductive and semantic orientation (Braun and Clarke, 2006, 2019). However, we did deviate from their guideline on certain points. Notably, we involved several members of the research team in the analyses, resulting in themes that reflect the shared interpretation of the broader research team.

In the first phase, we familiarized ourselves with the data by reading and re-reading the data. We made familiarization notes when applicable. In the second phase, we generated codes systematically: the interview transcripts were uploaded to ATLAS.ti version 9 (ALTAS.ti Scientific Software Development GmbH for Windows, Germany). One researcher and one research student (J.D.A. and D.M.) independently coded the first three interview transcripts for training purposes and to explore multiple perspectives on the data to enrich their individual coding. Following these initial three coded transcripts, consecutive transcripts were divided between these two authors for time efficiency. In the third phase, all generated codes were discussed, and initial themes were created together based on these codes. In the fourth phase, these initial themes were developed further and reviewed. In the fifth phase, these themes were reviewed, redefined, and named with additional members of the research team I.d.N., F.B.v.R., and N.M.v.M. by presenting all codes and suggested themes in a digital post-it session. Here, we generated six final themes: (i) personal considerations regarding fertility and fertility preservation in the past; (ii) external considerations regarding fertility and fertility preservation in the past; (iii) current vision on past considerations and decisions; (iv) current experiences and coping with infertility; (v) future family building; (vi) advice regarding fertility and fertility preservation decision-making. Finally, in the sixth phase, this article was drafted with in-depth input from all co-authors. Quotes related to the ideas within these themes were selected in consultation with the research team. Representative quotes were selected and translated by J.D.A. As a means of quality control, reversed translation of the quotes was performed by I.d.N. Furthermore, quotes were condensed by removing filler words while maintaining the concepts presented in the participants responses, as well as, adding context in brackets if anything was unclear in the quote.

SPSS version 20.0 (SPSS, Inc., Chicago, IL, USA) was used to summarize the participants’ demographics. All procedures performed in studies involving human participants were in accordance with the ethical standards of the institutional and/or national research committee and with the 1964 Helsinki declaration and its later amendments or comparable ethical standards. Informed consent was obtained from all individual participants included in the study.

### Researcher reflexivity

In qualitative research, the researchers themselves play an undeniable part. They bring their own unique identities, qualities, beliefs, and meanings to each aspect of the study (Olmos-Vega *et al.*, 2022). Therefore, it is important for the reader to be provided with information about the research team. We are a team of researchers and clinicians who have been involved in transgender reproductive research and healthcare for several years. All authors are white cisgender people from the Netherlands. We acknowledge the authors’ lack of diversity will influence this current study. For instance, we should be aware of heteronormative view on family building, also known as pronatalism (Bartholomaeus and Riggs, 2020). Furthermore, the interviewers were all affiliated to The Centre of Expertise on Gender Dysphoria which may influence the answers given by participants during the interview, even though the interviewers had no prior relationship with the participants nor knowledge of their medical history or -records and this was expressed as such by the interviewers at the beginning of the interview.

### Study group

Baseline characteristics (ethnicity, gender identity, sexual orientation, and relationship status) were participant self-descriptions discussed during the interview. With the participants permission, dates on starting gender-affirming treatments were retrieved from medical records after completing the interview if these dates were not recalled by the interviewee.

Of the 21 interviews conducted, eleven of the participants identified as male and ten of the participants identified as female. The mean age at the time of the interview was 29.2 (SD 3.1) years. Most of the participants identified as heterosexual (57%) and were single at the time of the interview (62%). Two people were stepparents at the time of the interview. For further details regarding the baseline characteristics, see Table 1.

**Table 1.** Baseline characteristics.

	All participants n = 21	Trans men n = 11	Trans women n = 10
Age at the time of interview	29.2 (3.1)	28.3 (2.2)	30.1 (3.9)
Age start puberty suppressants	15.3 (2.0)	15.5 (2.0)	14.9 (2.0)
Age start cross-sex hormones	16.8 (1.0)	16.9 (0.8)	16.5 (1.2)
Age at time of gonadectomy	19.6 (1.5)	19.1 (0.7)	20.2 (2.0)
Gender identity			
(trans)male	11 (52%)	11 (100%)	–
(trans)female	10 (48%)	–	10 (100%)
Ethnicity			
Dutch	19 (91%)	11 (100%)	8 (80%)
Missing	2 (9%)	–	2 (20%)
Sexual orientation			
Heterosexual	12 (57%)	8 (73%)	4 (40%)
Bisexual	5 (24%)	3 (27%)	2 (20%)
Homosexual	–	–	–
Asexual	1 (5%)	–	1 (10%)
Does not want to share	1 (5%)	–	1 (10%)
Missing	2 (9%)	–	2 (20%)
Current relationship status			
Single	13 (62%)	10 (91%)	4 (40%)
Relationship	6 (29%)	1 (9%)	5 (50%)
Married	1 (5%)	1 (9%)	–
Missing	1 (5%)	–	2 (20%)
Currently parent children	2 (9%)	1 (9%)	1 (10%)

Data are expressed in mean (SD) and numbers (%).

## Results

Following reflexive TA of the rich interview data, six themes were generated: (i) personal considerations regarding fertility and fertility preservation in the past; (ii) external considerations regarding fertility and fertility preservation in the past; (iii) current vision on past considerations and decisions; (iv) current experiences and coping with infertility; (v) future family building; (vi) advice regarding fertility and fertility preservation decision-making. These themes and their subthemes are presented in detail in the following paragraphs and an overview including supporting quotes is provided in Table 2. Within these themes and subthemes, there were no noticeable differences found between our participants based on their assigned sex at birth.

**Table 2.** Themes, corresponding subthemes, and illustrating quotes.

Themes		Corresponding subthemes		Supporting quotes	
1	Personal considerations regarding fertility and fertility preservation in the past	1.1	Gender affirmation versus fertility was not a choice	1. ‘I think I only ever saw it from one perspective. And that is not the children perspective, but the perspective of: this is the consequence of the choice to be happy yourself’.	Trans man (28 y/o)
				2. ‘Always difficult to speak of choices or decisions moments since I don’t feel that way. I don’t think my being transgender is a choice. I don’t think the steps I took for that were choices’.	Trans woman (30 y/o)
				3. ‘You know, you’re only concerned with one thing: surviving and making sure you can be yourself’.	Trans woman (28 y/o)
				4. ‘I was just really sure like, I want to be a boy and if I cannot have children as a boy, then I don’t want that’.	Trans man (25 y/o)
		1.2	Barriers related to gender dysphoria	5. ‘I had such intense dysphoria that… even the thought of semen coming out of me was so awful that I didn’t even want to talk about it’.	Trans woman (30 y/o)
				6. ‘I could probably never do that [retain uterus for future pregnancy], I think pregnancy is the pinnacle of femininity’.	Trans man (27 y/o)
		1.3	No desire for biological children	7. ‘There is more to it than just your DNA. It is an important part, but if you are there from the beginning of the pregnancy through birth and spend your entire life with a little one… well that is … so yes, there is more to it than just DNA’.	Trans man (27 y/o)
2	External considerations regarding fertility and fertility preservation in the past	2.1	Fertility counselling at the time	8. ‘In hindsight I feel it was quite neglected and it wasn’t talked about much’.	Trans woman (24 y/o)
		2.2	Role of treatment protocol and healthcare providers	9. ‘The trajectory at the VU always felt very laniary: puberty blockers, hormones, surgery, done’.	Trans woman (30 y/o)
				10. I felt like… not forced… But it was really presented to me as: this is what you want, this is what you need to do.	Trans woman (24 y/o)
		2.3	Experienced outside support or pressure	11. ‘I have quite a close friend group where we can discuss anything. Well, I am not the best talker, so I don’t talk about my feelings much, but those things were discussed’.	Trans man (26 y/o)
		2.4	Sterilization required by law for legal gender recognition	12. ‘It is very confronting to show your ID and to receive strange looks. For me that felt awful, so that’s why I wanted to proceed as quickly as possible’.	Trans man (26 y/o)
3	Current vision on past considerations and decisions	3.1	Reflection on fertility decisions made in the past	13. ‘I never felt a second of regret and I would go through it all again to be myself. However, the only thing I would do different is I would have frozen sperm’.	Trans woman (34 y/o)
		3.2	Influence of age on decision-making	14. ‘Can you ask a child? Not really. If you are honest, you cannot ask a 13/14 year old child if they want children later on. I think you cannot ask that, but unfortunately you must. And that is the problem… you have to’.	Trans woman (34 y/o)
				15. ‘But now that I am older, I am more aware you actually cannot have [biological] children anymore’.	Trans man (26 y/o)
4	Current experiences and coping with infertility	4.1	Emotions regarding infertility	16. ‘I never really thought about the fact I am infertile. Now that we are talking about it, I am like: am I infertile? Ooh yeah, I am infertile’.	Trans man (27 y/o)
				17. ‘It is silly to talk about, because I find it rude. But I noticed that the pregnancy of my sister was extremely confronting for me. I never cried as much as in those 9 months. Every ultrasound it was very clear to me this was never going to happen to me. When visiting the baby everyone said: “o she looks so much like you, she has your nose and your …” Every time that felt like a dagger. Because I could only think: this will never happen to me, no matter how close me and my future child may be’.	Trans woman (30 y/o)
		4.2	Coping with infertility	18. ‘I think in my case, for my peace of mind and quality of life, it is better to just accept that that [have biological children] naturally is not going to happen’.	Trans woman (30 y/o)
				19. ‘I always said I didn’t want kids, but maybe that is secretly because I thought I couldn’t have them anyway’.	Trans man (31 y/o)
		4.3	Experienced support	20. ‘I had to go to a psychologist for it because it really bothered me. All of my friends are having children and that gets to me’.	Trans man (28 y/o)
5	Future family building	5.1	Current desire for children	21. ‘Ultimately, I would have loved to have had children, that never changed. And I would love to have a biological child as well, very much so’	Trans woman (34 y/o)
		5.2	How to fulfil desire for children	22. ‘Of course, you have the option to adopt. I don’t think that is ever a bad idea actually since I feel you are also contributing something good to the world’.	Trans man (26 y/o)
				23. ‘I think out of naivety I am like: “oo maybe they will figure it out by then” Something like that’.	Trans woman (24 y/o)
		5.3	Importance of genetics	24. ‘Yes, it is not completely your own, but I would just love it as if it was my own child’.	Trans man (27 y/o)
6	Advice regarding fertility and fertility preservation decision-making	6.1	Advice to healthcare providers	25. ‘Make sure you [counsellor] don’t make it too heavy. Try to keep it light, everything else they are going through is already so much’.	Trans woman (28 y/o)
				26. ‘Actually, I think you should require all persons below 18 years of age to freeze something. Eggs, sperm, at least then you have something. We don’t have anything anymore’.	**Trans** man (28 y/o)
		6.2	Advice to transgender and gender diverse youth	27. ‘Get informed. And really think about it before you make a decision. Also ask for support from your friends and family if you can. Make sure you are 100% comfortable with your decision. Of course it is personal, but if you feel it is going too quick, just say so’.	Trans man (26 y/o)
				28. ‘I would maybe advise to put yourself out of your comfort zone and cross the threshold to do it. At least to keep your options open. Because you are not able to make such a decision at such a young age. And maybe you will want it later’.	Trans woman (26 y/o)

### Theme 1: personal considerations regarding fertility and fertility preservation in the past

When participants were asked to reflect on their past decision-making regarding fertility and fertility preservation in relation to their initiation of gender-affirming treatment, they displayed a remarkable recollection of the considerations they had during that time. Even though this decision-making process took place several years ago, nearly all participants said to vividly remember this period and the factors that influenced their choices.

#### Subtheme 1.1: gender affirmation versus fertility was not a choice

The participants’ perspectives shed light on the deeply rooted significance of their gender affirmation and the prioritization of their overall well-being over fertility preservation. The participants emphasized that the concept of ‘decision-making’ regarding reproductive abilities did not apply to them, as they saw their gender affirmation as an essential and non-negotiable aspect of their lives. They expressed a willingness to accept the consequences of becoming infertile as an unavoidable sacrifice to progress in their affirmation and achieve their primary goal [Table 2: Quotes 1, 2, and 3]. It became evident that time was of the essence for these individuals, as they expressed a strong desire to avoid any further delays in their affirmation. They perceived infertility as a price they were willing to pay, understanding that it was integral to reaching their true selves. This perspective demonstrated their unwavering commitment to their gender identity, regardless of the potential ramifications.

Moreover, the participants’ experiences of dysphoria extended beyond their physical selves and encompassed their gametes. They explained that using their biological gametes for reproduction would not align with their gender identity and would therefore be undesirable, highlighting the profound psychological and emotional impact of dysphoria on their sense of self and the importance of aligning all aspects of their lives with their affirmed gender [Table 2: Quote 4].

#### Subtheme 1.2: barriers related to gender dysphoria

The accounts of individuals who did contemplate fertility preservation shed light on the challenges they faced, particularly in relation to their experienced gender dysphoria. These participants highlighted specific barriers that made the process of fertility preservation seem burdensome and emotionally challenging. One notable barrier was the requirement of procedures such as masturbation for semen cryopreservation, which some individuals found to be too psychologically taxing or dysphoric to undertake [Table 2: Quote 5].

In addition, at the time of decision-making in adolescence, the idea of retaining reproductive organs to preserve the option for future reproduction, fertility preservation, or pregnancy, posed its own set of difficulties for some participants. They expressed discomfort or found it emotionally confronting to maintain these organs, as it conflicted with their affirmed gender identity [Table 2: Quote 6]. This reluctance to retain reproductive organs suggests the profound impact of gender dysphoria on their relationship with their bodies and the complexities they faced when considering fertility preservation options.

#### Subtheme 1.3: no desire for biological children

In addition to the participants who prioritized gender-affirming treatment over potential future parenthood, there were those who explicitly stated a lack of desire to become parents. For these individuals, the decision-making process regarding fertility preservation was straightforward. Their focus and aspirations centred around pursuing gender-affirming treatments, making the choice to forgo fertility preservation less complex and more aligned with their immediate goals. Parenthood was not a current priority for them.

On the other hand, some participants were uncertain about their future desires to become parents. They were at a crossroads, contemplating the possibility of parenthood but unable to commit to a definitive decision at that time. This ambivalence added an additional layer of complexity to their deliberations surrounding fertility preservation.

A common thread reported by participants was the perception that a biological relation to their future children was not of great importance to them. They prioritized other aspects of family building, rather than genetic ties [Table 2: Quote 7].

### Theme 2: external considerations regarding fertility and fertility preservation in the past

When participants reflected on their past decision-making processes, they highlighted the influence of various external factors in shaping their considerations regarding fertility and fertility preservation.

#### Subtheme 2.1: fertility counselling

According to guidelines and clinical protocols, individuals initiating gender-affirming treatment should have been informed about the potential negative impact of such treatments on their fertility. The majority of participants recalled receiving this information, indicating that healthcare providers adhered to these guidelines.

However, the depth and extent of discussions around fertility were found to be lacking [Table 2: Quote 8]. Participants expressed that while the topic of fertility may have been briefly mentioned during their interactions with healthcare providers, it was not adequately addressed or explored. Options for fertility preservation, which could have potentially mitigated the impact of gender-affirming treatment on their reproductive abilities, were either not discussed or only minimally touched upon. This limited engagement with the subject left participants feeling uninformed and ill-prepared to make well-informed decisions about their future fertility in hindsight.

#### Subtheme 2.2: role of treatment protocol and healthcare providers

In addition to participants’ reflections on their decision-making processes, some individuals conveyed their dissatisfaction with the treatment protocol at the gender clinic, describing it as strict, linear, and limiting. These participants perceived a lack of flexibility within the established treatment pathway. According to their accounts, transitioning from point A to point B involved a predetermined sequence of steps, and fertility preservation was not included as one of those steps. This rigid treatment pathway, as outlined by their healthcare providers, seemed to leave little room for discussion or exploration of alternative options [Table 2: Quote 10].

The participants’ expressions of frustration highlighted the potential limitations of a standardized treatment protocol that did not adequately address the diverse needs and concerns of transgender individuals regarding fertility.

#### Subtheme 2.3: experienced outside support or pressure

A significant number of participants had not only engaged in conversations about fertility with their healthcare providers, but also with their loved ones, including parents or friends. For some participants, these discussions provided valuable support regardless of the ultimate decision they made. They felt fortunate to have a network who were understanding, accepting, and supportive of their choices [Table 2: Quote 11].

However, it is important to note that not all participants experienced unequivocal support. Some individuals described feeling a certain degree of pressure from their parents to preserve gametes, who expressed a desire to become grandparents in the future. This external pressure of familial expectations added a level of complexity to their decision-making processes, as they had to reconcile their own desires and needs with the expectations and desires of their loved ones.

#### Subtheme 2.4: sterilization required by law for legal gender recognition

The decisions surrounding fertility and fertility preservation were made during a period when Dutch law mandated sterilization as a prerequisite for changing legal documents. Many participants expressed that their decision-making regarding gender-affirming treatment was not significantly influenced by this law. However, it is worth noting that some participants specifically recalled the pressing need they felt to change their legal documents as soon as possible. While their desire for gender-affirming treatment remained unchanged, the legal requirement may have influenced the timing of when they initiated treatment. This highlights the interplay between legal and personal considerations, as participants sought to navigate the complexities of aligning their legal gender identity with their affirmed gender and gender expression [Table 2: Quote 12].

### Theme 3: current vision on past considerations and decisions

#### Subtheme 3.1: reflection on fertility decisions made in the past

During the process of reflecting on their past considerations and decision-making, numerous participants expressed that, with the knowledge available today, they would make the same choice to initiate gender-affirming hormone treatment and undergo gonadectomy.

However, it is important to note that not all participants expressed this certainty in their past choices. Some individuals indicated that if they had the option, they would have chosen to freeze their gametes or retain their reproductive organs [Table 2: Quote 13]. This reveals a nuanced perspective, demonstrating that individuals’ views and desires regarding fertility and reproductive options may evolve over time, especially considering a developing and more accepting society.

#### Subtheme 3.2: influence of age on decision-making

Several participants acknowledged that their age at the time of initiating gender-affirming treatment played a significant role in their decision-making processes. Some individuals expressed the belief that they were too young when they first embarked on their gender affirmation journey to fully grasp the long-term impact of their decisions on future fertility [Table 2: Quote 14]. They recognized that the weight of such consequential choices requires a level of maturity and life experience that may not have been fully developed during their adolescent years.

These participants emphasized how their current age and subsequent maturity have shaped their current views on fertility and family building. They recognized that their perspectives and priorities have evolved over time, leading to a shift in how they perceive and value reproductive possibilities [Table 2: Quote 15]. With increased life experience and a deeper understanding of their own needs and aspirations, they now approach fertility considerations from a different vantage point than they did as adolescents.

The reflections shared by these individuals highlight the dynamic nature of decision-making and the influence of age and maturity on one’s perceptions of fertility. It underscores the importance of considering individual development and life experiences when making choices related to gender-affirming treatments and fertility preservation. As individuals grow older and gain a greater sense of self-awareness, their views on fertility and family building may evolve, leading to different considerations and priorities.

### Theme 4: current experiences and coping with infertility

During the interviews, participants shared their experiences of grappling with iatrogenic infertility and how it impacted their daily lives. Many varied experiences were shared.

#### Subtheme 4.1: emotions regarding infertility

Some participants described a neutral stance toward their infertility, stating that it was not a prominent concern in their lives, usually because they did not desire children. They expressed a sense of acceptance and did not mind discussing or acknowledging their infertility [Table 2: Quote 16]. However, there were others who strongly felt the weight of their infertility, experiencing emotions such as frustration, sadness, anger, and even regret. These individuals deeply mourned the loss of their reproductive capabilities and struggled with the emotional implications of their infertility [Table 2: Quote 17].

#### Subtheme 4.2: coping with infertility

For those participants who experienced negative emotions surrounding their infertility, coping strategies varied. Passive coping strategies, such as avoidance and acceptance, were commonly reported. Some individuals chose to avoid dwelling on their infertility or accepted it as an unchangeable reality [Table 2: Quotes 18 and 19]. On the other hand, some sought out support as an active coping strategy, see subtheme 4.3.

#### Subtheme 4.3: experienced support

The types of support participants received varied, with informal support being mentioned more frequently. Engaging in valued conversations with loved ones provided a sense of comfort and understanding. However, there were also individuals who sought out formal support through psychological therapy, recognizing the need for professional assistance in navigating their complex emotions related to infertility [Table 2: Quote 20].

### Theme 5: future family building

#### Subtheme 5.1: current desire for children

Participants held diverse visions and perspectives on current or future family building. While some expressed a lack of desire to have children, others expressed a strong desire to become parents [Table 2: Quote 21]. However, there were participants who remained uncertain about their parenting aspirations, citing reasons such as not having a partner or lacking financial stability at present.

#### Subtheme 5.2: how to fulfil desire for children

For participants with a desire to become parents, there was a range of options considered to fulfil this aspiration. Non-biological forms of parenthood, such as adoption, fostering, or using a donor/carrier, were mentioned as viable alternatives [Table 2: Quote 22]. These individuals were open to the idea of creating a family through avenues that do not rely on biological ties. Additionally, some participants expressed hope for future advancements in scientific techniques that could potentially offer them the opportunity for biological parenthood [Table 2: Quote 23].

#### Subtheme 5.3: importance of genetics

Similar to the previous subtheme, a significant majority of participants who expressed a desire to have children did not place a strong emphasis on biological relation. They were willing to accept or even prefer non-biological forms of parenthood [Table 2: Quote 24]. This indicates that for many individuals, the bond and experience of parenthood are prioritized over biological connections.

### Theme 6: advice regarding fertility and fertility preservation decision-making

#### Subtheme 6.1: advice to healthcare providers

When formulating advice for current healthcare providers, participants emphasized the importance of developing a customized counselling strategy specifically tailored for TGD individuals. They highlighted that a one-size-fits-all approach is not effective. To provide comprehensive and appropriate support, it was suggested to adapt counselling based on the information needs expressed by each individual, taking into account their unique circumstances and preferences. They also advised to try to keep it light [Table 2: Quote 25].

Furthermore, participants recommended that fertility counselling and preservation should be obligatory components of the care provided to TGD individuals. Recognizing the potential impact of fertility-related decisions on their future, participants stressed the need for healthcare providers to ensure that comprehensive information and options regarding fertility are made available to all individuals undergoing gender-affirming treatments [Table 2: Quote 26].

Additionally, participants expressed a sense of feeling lost or uncertain after completing their affirmation and treatment at the healthcare centre. To address this, they suggested the offering of continued guidance and resources to individuals even after they have completed their affirmation, as they may still require support in navigating various aspects of their lives.

#### Subtheme 6.2: advice to TGD youth

Participants offered advice for TGD youth who are currently considering gender-affirming treatment and making decisions about fertility and fertility preservation.

Primarily, participants emphasized the importance of seeking information and becoming well-informed about the potential impact of gender-affirming treatments on fertility. By gathering knowledge and understanding the options available, TGD youth can make more informed decisions that align with their individual needs and aspirations [Table 2: Quote 27].

Additionally, participants encouraged TGD youth to follow their hearts and make choices that feel authentic and true to themselves. This advice emphasizes the significance of self-discovery and self-advocacy, acknowledging that everyone’s journey is unique and personal.

Furthermore, participants emphasized the value of considering fertility preservation whenever possible. Recognizing the potential impact of gender-affirming treatments on fertility, participants recommended exploring options for fertility preservation to maintain the possibility of biological parenthood in the future. This advice highlights the importance of thinking ahead and making choices that take into account one’s future family-building aspirations [Table 2: Quote 28].

## Discussion

Based on TA of semi-structured interviews with adult transgender people, who are infertile due to gender-affirming treatment initiated in their adolescence, reflecting on their infertility, reproductive decision-making, and current family planning, we generated six main themes: (i) personal considerations regarding fertility and fertility preservation in the past; (ii) external considerations regarding fertility and fertility preservation in the past; (iii) current vision on past considerations and decisions; (iv) current experiences and coping with infertility; (v) future family building; (vi) advice regarding fertility and fertility preservation decision-making. These personal accounts underscore the intricate interplay between gender dysphoria and the decisions around fertility preservation in a time where restrictive legislation was present. The perceived burden and dysphoric nature of certain procedures and the discomfort associated with retaining reproductive organs highlight the multifaceted challenges that transgender individuals may encounter in navigating their reproductive health.

Theoretically, it is possible to postpone gender-affirming treatment until one’s (future) desire for children is fulfilled. However, for many TGD adolescents, it is imperative to start puberty blockers before reaching adulthood to prevent (further) development of secondary sex characteristics and be more satisfied with themselves and their bodies (de Vries *et al.*, 2014). This was also the case in our study. When reflecting on their past decision-making in regard to fertility, starting gender-affirming treatment took priority over potential future biological family building and this did not even feel like a choice at that time (Theme 1.1). Similar responses were also found in a recent study by Boguszewski *et al.* (2022), where authors conducted a focus group and individual interviews with 25 TGD adolescents and young adults and six of their parents learning about the effects of gender-affirming treatments on fertility as well as the process of making a fertility preservation decision. Here, participants described the urgent need to begin gender-affirming treatments as time-sensitive treatment which precluded fertility preservation resulting in the theme ‘External Constraints Limit Choices’. A similar theme, ‘Focus on Transition’ was also identified by Kerman *et al.* (2021), where they conducted 23 semi-structured interviews with TGD youth (median 17 years old) on fertility and future family building. Comparable findings were also described in a recent quantitative survey study by de Nie *et al.* (2023a), where Dutch adult TGD people reflected on their fertility decisions made in adolescence in a time where legislation mandated sterilization. Here, 67% of 207 survey participants reported to have had prioritized their gender-affirming treatment over staying fertile while making these decisions.

When evaluating the role of fertility counselling and clinical care protocols at the time (Themes 2.1, 2.2, and 2.4), it is important to note that the absence of explicit consideration for fertility preservation within the established pathway may have left participants feeling constrained or deprived of opportunities to explore and make informed decisions about their reproductive future. Thankfully, this has changed significantly since our participants initiated their gender-affirming treatment. Since legislation changed in 2014, our centre’s clinical practice is in line with the current Standards of Care, where fertility counselling by a fertility specialist or gynaecologist is offered to TGD people prior to initiating puberty blockers, GAHT and GAS (Coleman *et al.*, 2022). Fertility preservation options are offered and covered by Dutch healthcare insurance. This is significant, since the cost of fertility preservation treatment is often mentioned as a barrier in other studies (Chen *et al.*, 2017; Vyas *et al.*, 2021; Boguszewski *et al.*, 2022). The advice of customized fertility counselling to TGD youth (Theme 6.1) is already being implemented as much as possible at our centre.

We considered the prior legal requirement (Theme 2.4) may have had an impact on the decision-making processes of TGD individuals during that time. However, many participants expressed that their decision-making regarding gender-affirming treatment was not significantly influenced by this law. Nonetheless, it is worth noting that some participants recalled feeling a pressing need to change their legal documents as soon as possible, adding an additional layer of complexity to their decision-making process. This highlights the challenges of navigating such choices, especially under the added pressure experienced at that age. Overall, this suggests a strong commitment to their gender identity and a prioritization of their own well-being over possible future parenthood, similar to de Nie *et al.* (2023a).

When reflecting on past considerations, the influence of age was an important factor (Theme 3.2) in the decision-making process. Some of our participants expressed they were too young or not mature enough at the time to make decisions regarding their fertility. This finding is in line with another study by Kerman *et al.* (2021). This qualitative interview study in TGD adolescents, identified a main theme ‘age disconcordance’ where participants also expressed not to have an interest in future family building, specifically due to their age. These participants also acknowledged their views on this topic may change over time. This development over time is also seen in the general population (Guzzo *et al.*, 2019). In clinical practice, determining the mental competence in regard to fertility will remain a point of discussion due to the young age of patients starting gender-affirming hormone treatment and subsequent infertility. Especially since TGD adolescents are capable to give informed consent in regard to starting puberty blockers (Vrouenraets *et al.*, 2021).

Current feelings on infertility (Theme 4.1) varied greatly in our participants. These personal experiences shed light on the diverse emotional responses to iatrogenic infertility and highlight the importance of coping strategies and support systems. Participants demonstrated a range of coping mechanisms, with some finding solace in acceptance while others actively sought support from informal or formal sources. These findings underscore the significance of providing personalized comprehensive emotional support and resources to individuals navigating the emotional challenges of infertility within the context of gender-affirming treatments. These feelings were also reported in the previously mentioned study by de Nie *et al.* (2023a). When asked how participants felt about not having children, 42% of 207 survey participants indicated to not have problems with their infertility at all and 31% indicated to struggle with this in varying degrees (de Nie *et al.*, 2022). Negative feelings in regard to infertility were also found in other cohorts. For instance, a study by Armuand *et al.* (2014) described a cohort of infertile cancer survivors, where an unfulfilled desire to have children was associated with worse mental health. Moreover, the psychosocial effect of infertility in cis gender couples has been highly documented as well (Moura-Ramos *et al.*, 2011). Neutral or accepting feelings on infertility were also discussed. These feelings could also be the result of a successful coping strategy (Theme 4.2).

### Strengths and limitations

Being one of the first qualitative studies to explore the vision of infertile transgender people who started gender-affirming treatment during adolescence, the major strength of this study is its unique study cohort and its rich contribution to the limited data on this subject in this particular research group. This research highlights the enduring impact of the choices made by a patient as well as by their parents and caregivers during the initiation of gender-affirming treatment, specifically during a time where restrictive legislation was present. It offers valuable insights into the long-lasting significance of these decisions and emphasizes the need for comprehensive support and informed decision-making processes in the realm of fertility and fertility preservation for transgender individuals today. Furthermore, the nature of qualitative research entails we gain a deeper understanding of people’s views, experiences, and motivations (Sardana *et al.*, 2023). This data may help improve current fertility counselling in TGD youth to aid them in their decision-making in regard to gender-affirming treatment and (in)fertility.

However, this study is not without its limitations. We interviewed a select group of individuals, which may introduce selection bias (Collier and Mahoney, 1996). Although we ensured equal distribution of trans masculine and feminine individuals by considering assigned sex at birth during recruitment, we unintentionally ended up with a sample consisting of individuals with a binary gender identity. Additionally, participants’ relationships with our gender clinic may have influenced their decisions to participate, with some harbouring resentment for undergoing sterilizing treatment while others feeling gratitude for receiving gender-affirming treatment. Furthermore, individuals with strong opinions on this topic may have been more inclined to participate than those with a neutral stance. Similarly, individuals who found the one-on-one interview structure too confronting may have declined participation. These aspects of selection bias may have influenced the extent to which the experiences of our sample reflect those of the broader TGD population who are currently infertile due to gender-affirming treatment initiated in adolescence. Since selection bias is inherit to qualitative research, we aimed to mitigate this by utilizing a large and diverse sample.

Other forms of bias may also play a role in this study. For one, recall bias since participants were asked to reflect on a period many years ago (Sedgwick, 2012). Choice supportive bias may also be present, this is a form of bias where people retroactively attribute positive feelings to their decisions as a coping strategy to be at peace with these decisions and avoid feelings of regret (Lind *et al.*, 2017). This may have led to more positive accounts described by our participants.

Furthermore, it should be noted that this sample is not representative of the current situation, since legislation in the Netherlands has been changed in 2014 to no longer require infertility to change sex on legal documents. Decisions regarding GAS with permanent implications for fertility, can since be made with greater intrinsic consideration. Most of the young people who began transitioning after 2014 are not yet at the age where they are most likely to want children. Therefore, the group of people we studied is the only group to provide information and advice on fertility matters for the current population. Following the completion of our data analysis, Braun and Clarke (2023) published an article outlining best practices and potential pitfalls in reflexive TA. This publication prompted us to reassess certain aspects of their method that we had interpreted differently. For instance, the use of multiple researchers to generate themes. While the use of two coders is identified as a common pitfall in reflexive TA, it is important to clarify that our decision to employ two coders was primarily driven by the need for training purposes of a research student and to enhance time efficiency, rather than the purpose of increasing reliability or achieving code consensus which is generally discouraged by Braun and Clarke. While this insight cannot be rectified in retrospect, we prioritize transparency, which is why we have explicitly addressed our modified method throughout the manuscript.

## Conclusions

To our knowledge, the current study is the first to qualitatively assess reproductive decision-making and current family-building desires in TGD people who were required by Dutch law to undergo sterilization prior to gender affirmation treatment. This semi-structured interview study generated six main themes encompassing the many topics discussed when reflecting on adolescent decision-making in regard to starting gender-affirming treatment and (in)fertility, current experiences with infertility, coping strategies, and future family planning. By sharing their experiences, the participants shed light on the lived realities of those facing the intersection of gender identity, gender dysphoria, and fertility preservation decisions. The present study shows, in the context of since abolished legislation, even though medical affirmation takes priority over fertility(preservation) for many TGD adolescents, some may develop a strong desire for genetic offspring in adulthood. And even though some transgender people currently express negative emotions such as anger, sadness, and regret related to their infertility, they have found ways to cope and most of these people would still make the same decisions regarding their gender-affirming treatment today. Our findings contribute to the limited data on long-term effects of gender-affirming treatment started in adolescence and reaffirm the need for fertility (preservation) counselling prior to gender-affirming treatment which may negatively affect a person’s fertility and family-building options in the future.

## Supplementary Material

deae155_Supplementary_Data

## Data Availability

The data supporting this article will not be made available for sharing due to ethical and privacy considerations. Participants in the study did not provide consent for their anonymized transcripts to be accessible to others, and given the qualitative nature of the research design, there is a potential risk of re-identifying a participant, which could compromise their privacy.
