# Supplementary material for: Transgender persons’ view on previous fertility decision-making and current infertility: a qualitative study
Source: Hum Reprod. 2024 Jul 15;39(9):2032–42. doi: 10.1093/humrep/deae155 (PMC11373325; doi:10.1093/humrep/deae155)
Supplement: deae155_Supplementary_Data [file deae155_supplementary_data.pdf]

OXFORD
human reproduction

Human Reproduction, 2024, 1–1
https://doi.org/10.1093/humrep/deae155
Supplementary Data

Supplementary Table S1. Semi-structured interview script.

Phases	Core questions						
Prior/during medical affirmation	*Did you want to be a parent prior to your medical affirmation?*	*Were options regarding fertility (preservation) discussed prior to medical affirmation? If yes, when, how, and by whom?*	*Was the information provided sufficient in your opinion?*	*How did you end up deciding on fertility (preservation)? What were significant arguments either way?*	*Did your parents or other healthcare providers play a role in your decisions? Anyone else? If yes, how?*	*How did the legal requirement for sterilization play a role in your decision-making regarding fertility (preservation)?*	*How did you experience the knowledge of becoming infertile due to a medical treatment?*
Following medical affirmation	*Has your opinion on wanting to parent children change or fluctuate since treatment? If yes, when, and why?*	*Have your feelings about becoming infertile change or fluctuate since treatment? If yes, when, and why?*					
Currently	*How do you feel about parenting children currently or in the future?*	*How do you feel about being infertile? And how do you cope with these feelings?*	*Does the 2014 change in legislation influence your feelings about becoming infertile?*	*Looking back on your decisions regarding fertility, would you have done anything differently if you could?*	*Have you explored options surrounding non-biological parenting? If yes, how satisfied are you with those options?*	*Looking back, do you feel you were old enough to make decision regarding fertility? If no, what age would be better suited?*	

© The Author(s) 2024. Published by Oxford University Press on behalf of European Society of Human Reproduction and Embryology.
This is an Open Access article distributed under the terms of the Creative Commons Attribution License (https://creativecommons.org/licenses/by/4.0/), which permits unrestricted reuse, distribution, and reproduction in any medium, provided the original work is properly cited.
Human Reproduction, 2024, 00, 1–12
https://doi.org/10.1093/humrep/deae155
Original Article
